# Prognostic value of intratumoral metabolic heterogeneity on F-18 fluorodeoxyglucose positron emission tomography/computed tomography in locally advanced cervical cancer patients treated with concurrent chemoradiotherapy

**DOI:** 10.18632/oncotarget.18769

**Published:** 2017-06-28

**Authors:** Gun Oh Chong, Won Kee Lee, Shin Young Jeong, Shin-Hyung Park, Yoon Hee Lee, Sang-Woo Lee, Dae Gy Hong, Jae-Chul Kim, Yoon Soon Lee

**Affiliations:** ^1^ Department of Obstetrics and Gynecology, Kyungpook National University Medical Center, School of Medicine, Daegu, Republic of Korea; ^2^ Medical Research Collaboration Center in KNUH, Kyungpook National University, School of Medicine, Daegu, Republic of Korea; ^3^ Department of Nuclear Medicine, Kyungpook National University Medical Center, School of Medicine, Daegu, Republic of Korea; ^4^ Department of Radiation Oncology, Kyungpook National University Medical Center, School of Medicine, Daegu, Republic of Korea

**Keywords:** intratumoral metabolic heterogeneity, locally advanced cervical cancer, 18F-FDG PET/CT, concurrent chemoradiotherapy, prognosis

## Abstract

**Objective:**

To evaluate the prognostic value for predicting tumor recurrence of intratumoral metabolic heterogeneity and traditional quantitative metabolic parameters on pre-treatment F-18-fluorodeoxyglucose positron emission tomography/computed tomography (18F-FDG PET/CT) in patients with locally advanced cervical cancer treated with concurrent chemoradiotherapy (CCRT).

**Materials and Methods:**

Ninety-three patients with biopsy-proven cervical cancer and treated with CCRT (FIGO stage IIB-IV) were enrolled in this study. The traditional metabolic parameters of the primary tumor, regional lymph node, and whole body (maximum standardized uptake value [SUVmax], metabolic tumor volume [MTV], and total lesion glycolysis), and intratumoral heterogeneity factor (HF) were measured on pre-treatment 18F-FDG PET/CT images. Univariate and multivariate analyses for disease-free survival (DFS) were performed using clinical and metabolic parameters. The additional HF prognostic value was evaluated by means of time-dependent receiver operating characteristic curve, integrated discrimination improvement, and net reclassification improvement.

**Results:**

On multivariate analysis, nodal SUVmax (hazard ratio 3.60; 95% CI, 1.66–7.85; *p* = 0.0012) and whole body MTV (WBMTV; hazard ratio 3.15; 95% CI, 1.17–8.53; *p* = 0.0236) were significant prognostic factors for DFS. When HF was combined with nodal SUVmax and WBMTV, a significant improvement in discrimination for recurrence was observed compared with nodal SUVmax alone (area under curve 0.817 vs. 0.732; *p* = 0.0028).

**Conclusions:**

HF did not show superiority over traditional metabolic parameters. However, when HF was combined with nodal SUVmax and WBMTV, the predictive value for tumor recurrence improved. Therefore, HF may be a useful additional prognostic biomarker to improve the prognostic value of traditional metabolic parameters on 18F-FDG PET/CT.

## INTRODUCTION

For patients with locally advanced cervical cancer (stage IIB to IV), concurrent chemoradiotherapy (CCRT) using a cisplatin-based regimen has become the standard treatment [1, 2]. However, although the contribution of CCRT to an improvement in survival outcomes of cervical cancer has been well confirmed, the outcomes of patients with locally advanced cervical cancer have been unsatisfactory [3]. The Advanced International Federation of Gynecology and Obstetrics (FIGO) stage, larger tumor size, and presence of lymph node metastasis were reported as negative prognostic factors for cervical cancer treated with CCRT [4, 5].

Currently, F-18 fluorodeoxyglucose positron emission tomography/computed tomography (^18^F-FDG PET/CT) is being widely used to detect lymph node involvement, distant metastasis, and recurrent disease in cervical cancer [6]. Moreover, several reports have demonstrated that metabolic parameters of primary tumor and regional lymph node on ^18^F-FDG PET/CT may be prognostic biomarkers for the prediction of disease recurrence in patients with locally advanced cervical cancer [7–9]. Recently, we compared the prognostic value of metabolic parameters of primary tumors and regional lymph nodes and found that nodal maximum standardized uptake value (SUVmax) according to pre-treatment ^18^F-FDG PET/CT was the most powerful biomarker to predict tumor recurrence [10]. There is emerging evidence that intratumoral metabolic heterogeneity on pre-treatment ^18^F-FDG PET/CT might be a predictor of tumor recurrence after CCRT in patients with lung cancer, esophageal cancer, and head and neck cancer [11–13]. Similarly, several studies were performed to evaluate the prognostic value of intratumoral metabolic heterogeneity on pre-treatment ^18^F-FDG PET/CT in patients with cervical cancer treated with CCRT [14, 15]. Due to the limited number of previous studies on intratumoral metabolic heterogeneity on pre-treatment ^18^F-FDG PET/CT, and the difficulty in evaluating it because of the small size of the primary tumor, its exact role in predicting tumor recurrence has not been fully investigated in patients with locally advanced cervical cancer. Moreover, to date, the superiority of intratumoral metabolic heterogeneity over standard quantitative metabolic parameters, including SUVmax, metabolic tumor volume (MTV), and total lesion glycolysis (TLG), has not been demonstrated.

The aim of the present study was to compare the prognostic value of intratumoral metabolic heterogeneity for the prediction of tumor recurrence with that of standard quantitative metabolic parameters on pre-treatment ^18^F-FDG PET/CT in patients with locally advanced cervical cancer treated with CCRT, and to assess its correlation with traditional metabolic parameters.

## MATERIALS AND METHODS

### Patients

For this study, we enrolled 93 patients with biopsy-confirmed cervical cancer treated with CCRT between September 2005 and August 2014. Retrospective data collection and analysis were approved by the Institutional Review Board of Kyungpook National University Medical Center. The need for informed consent was waived due to the retrospective design of the study. The patients were staged according to the FIGO staging system. All patients had undergone ^18^F-FDG PET/CT for initial diagnosis, staging, and radiotherapy planning. Patients who exhibited evidence of distant metastatic disease or history of previous surgery, radiotherapy, or chemotherapy were excluded from the study.

The clinical and pathological parameters were reviewed and retrieved, including age, serum squamous cell carcinoma antigen, FIGO stage, histology, primary tumor size, and presence of pelvic and paraaortic lymph node metastasis.

### Treatment

All patients were treated with a combination of external beam radiotherapy and high-dose-rate intracavitary brachytherapy with curative intent. External beam radiotherapy was delivered to the whole pelvis using 10 MV photons with customized shielding in 1.8 Gy daily fractions, five times a week, for a total dose of 45 Gy. A four-field box technique was used. The superior border was at the L4–L5 vertebral level. The inferior border was at the bottom of the obturator foramen or 2–3 cm below the lowest extent of the cervical or vaginal disease. The lateral borders were placed 2 cm lateral to the inner bony margins of the true pelvis. For the lateral fields, the anterior border included the symphysis pubis, and the posterior border was the S2-3 interspace. For patients with paraaortic nodal involvement, the superior border extended to the T12-L1 interspace. Boost external beam radiotherapy of 10 Gy in five fractions was indicated for patients with parametrial involvement and/or nodal metastases. High-dose-rate intracavitary brachytherapy was initiated after an external beam radiotherapy dose of 39.6 Gy. Intracavitary brachytherapy was delivered twice a week in five fractions with a fractional dose of 6 Gy at point A. At the end of parametrial and nodal boost external beam radiotherapy, ^18^F-FDG PET/CT or CT scans were performed. In cases of residual pelvic lymphadenopathy, an additional boost external beam radiotherapy of 4–10 Gy was applied. As a result, a median of 65 Gy (range, 59–65 Gy) was irradiated for gross residual lymphadenopathy after parametrial and nodal boost. Weekly cisplatin at a dose of 40 mg/m^2^ was administered during radiotherapy. The first course of cisplatin was administered on day 1 of radiotherapy.

## FDG PET/CT IMAGE ACQUISITION

All patients fasted for at least 6 hours and their blood glucose levels were checked before the administration of FDG. Patients with blood glucose levels higher than 150 mg/dL were rescheduled for examination, and treatment was administered to maintain a blood glucose concentration of < 150 mg/dL in all participants. Patients received intravenous injections of approximately 8.1 MBq of FDG per kg of body weight and were advised to rest for 1 hour before the acquisition of the FDG PET/CT image. FDG PET/CT scans were performed using a Reveal RT-HiREZ 6-slice CT apparatus (CTI Molecular Imaging, Knoxville, TN, USA) and a 16-slice CT Discovery STE apparatus (GE Healthcare, Milwaukee, WI, USA). Before the PET scan, for attenuation correction, a low-dose CT scan was obtained without contrast enhancement from the skull base to the thigh with the patient in a supine position and breathing quietly. PET scans with a maximum spatial resolution of 6.5 mm (Reveal PET/CT) and 5.5 mm (Discovery PET/CT) were also obtained from the skull base to the thigh at 3 minutes per bed position. The PET images obtained using the Reveal PET/CT and Discovery PET/CT scanners were reconstructed with a 128 × 128 matrix, an ordered-subset expectation maximum iterative reconstruction algorithm (4 iterations; 8 subsets), a Gaussian filter of 5.0 mm, and a slice thickness of either 3.0 mm (Reveal PET/CT) or 3.27 mm (Discovery PET/CT).

### Image analysis

Image display and analysis were achieved using the volume viewer software on an Advantage Workstation 4.5 (GE Medical Systems, Milwaukee, WI, USA), which provides a convenient and automatic method to delineate the volume of interest using an isocontour threshold method based on the SUV. For each patient, the SUVmax was designated as the highest SUV of the primary tumor and regional lymph nodes, and MTV and TLG were obtained by adding the values of the primary tumor and all regional lymph nodes. The SUVmax was obtained using the following formula: *SUVmax = maximum activity in the region of interest (MBq/g)/(injected dose [MBq]/body weight [g])*. The mean SUV of the mediastinal background plus two standard deviations was used as the threshold to calculate MTV automatically. The TLG of a lesion was calculated as the MTV multiplied by the SUVmean. The MTV and TLG of the regional lymph nodes were defined as the sum of those parameters for each lymph node. Semi-quantitative and volumetric analyses of the primary tumor were performed using the volume viewer software on a GE Advantage Workstation 4.3 (GE Healthcare, Milwaukee, WI, USA).

To obtain intratumoral metabolic heterogeneity, the method of a previous study was followed [16]. The intratumoral metabolic heterogeneity was represented by the heterogeneity factor (HF), which was determined for each patient as follows: a region of interest was manually drawn to include the primary tumor and a surrounding region of normal tissue (normal background). The tumor volume was determined with a series of SUV thresholds (e.g., 40%, 50%, 60%, 70%, and 80% of SUVmax) using the semi-automatic software of the workstation (Advantage Workstation 4.3, GE Healthcare). We excluded the values < 40% from the heterogeneity analysis because a previous study reported that the minimal threshold that represents the actual tumor volume was 40%, while the values < 40% included too much normal tissue background activity [17]. In addition, values > 80% were also excluded because the volumes were small and the partial volume effect was pronounced [18]. A volume-threshold function of the tumor was acquired by plotting thresholds to volumes (Supplementary Figure 1). A linear regression analysis was performed and the HF was calculated by finding the derivative (dV/dT) of the volume-threshold function for each tumor. Subsequently, the HF values were modified into absolute values so that all resulting values were positive. The more positive the factor, the more heterogeneous the tumor. It took approximately 1 minute to obtain the intratumoral metabolic heterogeneity for each patient.

### Clinical follow-up

Clinical follow-ups of patients were performed every 3 months until 2 years, every 6 months after 2 years and up to 5 years, and annually thereafter. After completion of the treatment, ^18^F-FDG PET/CT was performed for all patients. Failure was defined as biopsy-proven recurrence or documentation of progression of disease on serial imaging studies. Failure patterns were divided into four groups: (1) none, (2) isolated local failure that included the central pelvis and/or pelvic lymph nodes, (3) distant failure that included paraaortic and supraclavicular lymph nodes, and (4) combined local and distant failure.

### Statistical analysis

Continuous data were expressed as means ± standard deviation and categorical data were presented as frequencies and percentages. The time to event was calculated as the time interval from the date of diagnosis to the date of the first clinical or imaging findings that suggested disease recurrence. The differences between subsets were evaluated by a Student's *t*-test and differences between proportions were compared with a chi square test. Spearman's correlation analysis was used to clarify the relationships between the PET parameters (HF, SUVmax, MTV, and TLG). The Contal and O’Quigley technique was used to select the cutoff value for the ^18^F-FDG uptake values of the primary tumor, regional lymph nodes, and HF. To distinguish between high and low risk groups, the optimal cutoff value was determined by an algorithm of maximization of the hazard ratio [19–20].

The additional prognostic value of HF was evaluated by means of receiver operating characteristic (ROC), integrated discrimination improvement, and net reclassification improvement [21]. To determine its exact disseminative ability, the cut-off value was determined using time-dependent ROC curves and the corresponding integrated area under the curves was calculated to assess the predictive accuracy for tumor recurrence. The mean difference and 95% confidence interval (CI) of the integrated area under the curves was tested as previously described [22].

A univariate Cox proportional hazards model was used to determine the hazard ratios of prognostic factors for disease-free survival (DFS). A forward stepwise multivariate Cox proportional hazards model was used to assess the potential independent effects of prognostic factors for DFS, and an estimated hazard ratio (HR) with 95% CI was presented. Survival curves of prognostic factors were estimated using the Kaplan-Meier method and the differences between subgroups were compared by a log-rank test. To divide the subgroups, weights factor for each parameter were calculated using estimation coefficients of the Cox proportional hazards model. Lastly, the Contal and O’Quigley technique was used to classify patients into subgroups.

SAS version 9.4 (SAS Institute Inc, Cary, NC) was used for statistical analysis. Time-dependent ROC and integrated AUC were performed by R for windows (version 3.3.1, R Foundation for Statistical Computing, Vienna, Austria) with survivalROC and risksetROC packages. A *p*-value less than 0.05 was considered statistically significant.

## RESULTS

### Clinical features and treatment outcomes

The clinical characteristics of the study participants are listed in Table 1. The mean age was 53.1 ± 12.6 years. The predominant FIGO stage was IIB. Fifty-three patients (57.0%) had pelvic nodal metastases and thirteen patients (14.0%) had paraaortic nodal metastases. After a median follow-up of 55 months (range 9–124 months), 29 patients (31.2%) had recurrence while 21 patients (22.6%) had died due to disease progression. Of the 29 patients who experienced disease recurrence, 12 patients had local recurrence only, 13 patients had distant recurrence only, and four patients had both local and distant recurrence.

**Table 1:** Clinicopathologic characteristics and PET metabolic parameters of patients with and without recurrence

Variables	All (*n* = 93)	No recurrence (*n* = 64)	Recurrence (*n* = 29)	*p*-value
Age (years)	53.1 ± 12.6	54.7 ± 12.5	49.6 ± 12.5	0.069
FIGO stage (*n*, %)				
IIB	75 (80.6)	56 (87.5)	19 (65.5)	0.001
IIIA1	4 (4.3)	4 (6.2)	0 (0)	
IIIA2	8 (8.6)	3 (4.7)	5 (17.2)	
IIIB	5 (5.4)	0 (0)	5 (17.2)	
IV	1 (1.1)	1 (1.6)	0 (0)	
Histology (*n*, %)				
Squamous cell carcinoma	85 (91.4)	60 (93.7)	25 (86.2)	0.396
Adenocarcinoma	8 (8.6)	4 (6.2)	4 (13.8)	
Tumor size (cm)	4.5 ± 1.6	4.3 ± 1.4	5.1 ± 1.9	0.014
Lymph node metastasis (*n*, %)				
Pelvic	53 (57.0)	33 (51.6)	20 (69.0)	0.116
Paraarotic	13 (14.0)	5 (7.8)	8 (27.6)	0.011
SCC antigen (ng/mL)	20.6 ± 37.2	16.5 ± 38.8	26.2 ± 30.4	0.263
Metabolic PET parameters				
Primary tumor SUVmax	14.9 ± 7.6	14.8 ± 8.6	15.0 ± 4.7	0.946
Primary tumor MTV (cm^3^)	80.1 ± 96.2	63.4 ± 56.0	116.9 ± 146.0	0.012
Primary tumor TLG	591.1 ± 804.2	497.1 ± 654.2	798.5 ± 1047.6	0.094
Nodal SUVmax	4.1 ± 6.5	3.1 ± 5.0	6.5 ± 8.7	0.020
Nodal MTV (cm^3^)	8.3 ± 25.9	3.5 ± 19.1	7.2 ± 43.8	0.006
Nodal TLG	37.8 ± 139.0	14.5 ± 37.7	89.1 ± 237.3	0.016
WBMTV (cm^3^)	88.4 ± 106.8	66.8 ± 58.3	136.0 ± 162.2	0.003
WBTLG	628.9 ± 842.8	511.6 ± 675.4	887.6 ± 1098.6	0.046
HF	1.072 ± 1.436	0.781 ± 0.614	1.714 ± 2.303	0.003

FIGO = International Federation of Gynecology and Obstetrics; MTV = metabolic tumor volume; SCC = squamous cell carcinoma; SUVmax = maximum standardized uptake value; TLG = total lesion glycolysis; WBMTV = whole body metabolic tumor volume; WBTLG = whole body total lesion glycolysis; HF; heterogeneity factor.

### Correlation between HF and PET metabolic parameters

**Figure 1:**
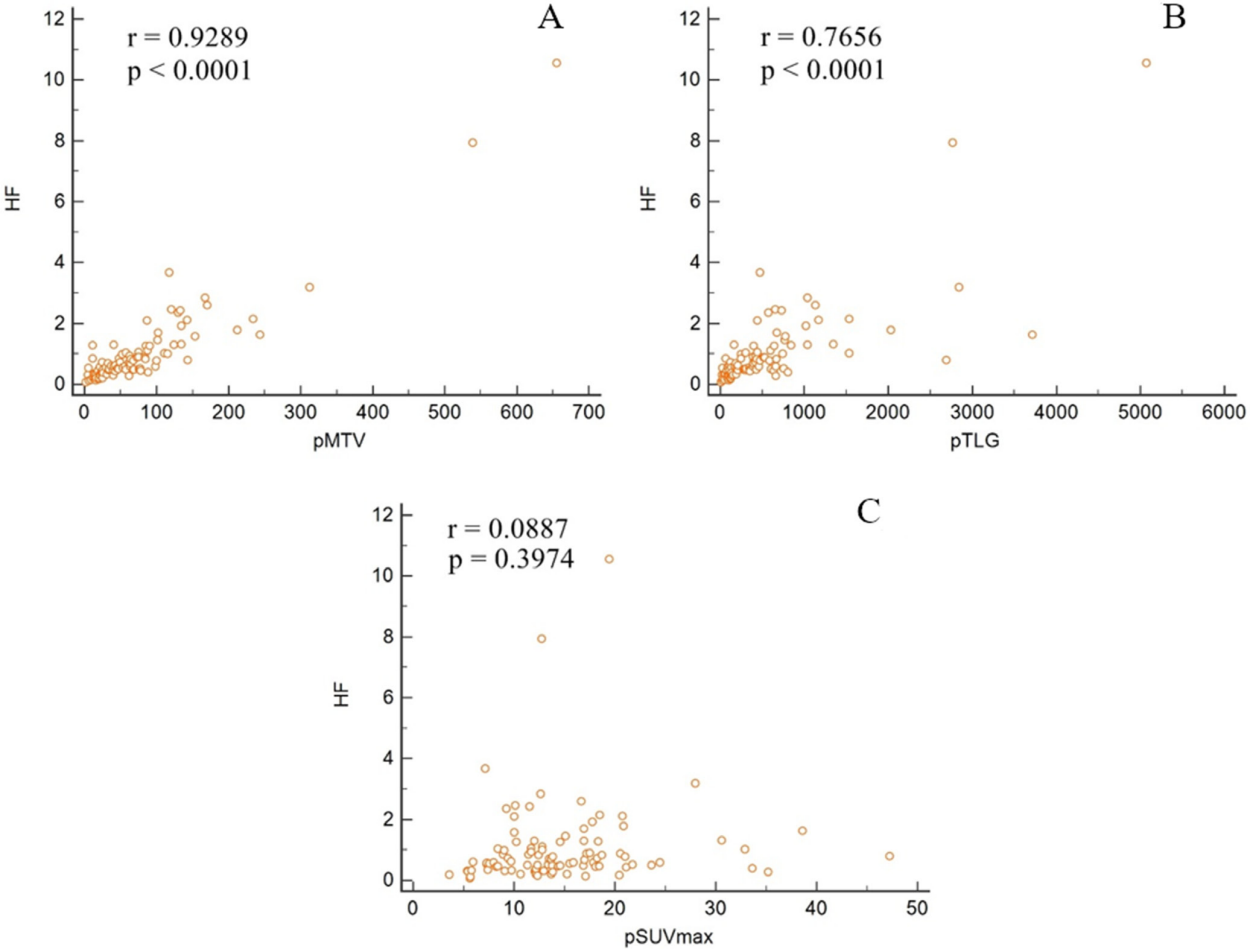
Correlation between heterogeneity factor and traditional metabolic parameters

In the analysis of the correlation between HF and PET metabolic parameters, there was a significant correlation between HF and primary tumor MTV (r = 0.9289, *p* < 0.0001) and HF and primary tumor TLG (r = 0.7656, *p* < 0.0001). However, HF did not correlate with primary tumor SUVmax (r = 0.0887, *p* = 0.3974; Figure 1) .

### Survival analyses

The mean primary tumor HF in the recurrent group was significantly higher than that in the non-recurrent group (Table 1). However, primary tumor SUVmax and TLG were not significantly different between the non-recurrent and recurrent groups. The nodal metabolic parameters of the recurrent group were significantly higher compared to those of the non-recurrent group (Table 1). Whole body MTV (WBMTV) and whole body TLG (WBTLG) were significantly higher in the recurrent group compared to those of the non-recurrent group (Table 1).

The Contal and O’Quigley technique demonstrated that an HF of 0.68 was the optimal cut-off for predicting tumor recurrence (*p* = 0.009; sensitivity, 70.0%; specificity, 60.9%; AUC, 0.659; standard error, 0.061). The univariate analysis confirmed the following significant prognostic factors for DFS (Table 2): age (HR: 2.20); tumor size (HR: 2.81); FIGO stage (HR: 2.50); paraaortic nodal metastasis (HR: 3.15); serum squamous cell carcinoma antigen (HR: 3.37); primary tumor SUVmax (HR: 3.10); primary tumor MTV (HR: 3.78); primary tumor TLG (HR: 3.50); nodal SUVmax (HR: 4.79); nodal MTV (HR: 3.65); nodal TLG (HR: 3.34); WBMTV (HR: 4.50); WBTLG (HR: 4.84); and HF (HR: 2.86).

**Table 2:** Univariate and multivariate analyses of clinical variables and quantitative metabolic parameters for recurrence

Variables	Univariate analysis			Multivariate analysis		
	HR	95% CI	*p*-value	HR	95% CI	*p*-value
Age	2.20	1.06–4.59	0.0347			
Tumor size	2.81	1.24–6.35	0.0131			
FIGO Stage	2.50	1.14–5.49	0.0229			
Pelvic lymph node	1.87	0.85–4.11	0.1198			
Paraaortic lymph node	3.15	1.38–7.18	0.0064			
SCC antigen	3.37	1.60–7.10	0.0014			
Primary tumor SUVmax	3.10	1.32–7.28	0.0093			
Primary tumor MTV	3.78	1.54–9.31	0.0038			
Primary tumor TLG	3.50	1.49–8.21	0.0040			
Nodal SUVmax	4.79	2.25–10.20	< 0.0001	3.60	1.66–7.85	0.0012
Nodal MTV	3.65	1.75–7.61	0.0006			
Nodal TLG	3.34	1.57–7.10	0.0017			
WBMTV	4.50	1.72–11.82	0.0023	3.15	1.17–8.53	0.0236
WBTLG	4.84	1.68–13.91	0.0035			
HF	2.86	1.30–6.28	0.0091			

CI = confidence interval; FIGO = International Federation of Gynecology and Obstetrics; HR = hazard ratio; MTV = metabolic tumor volume; SCC = squamous cell carcinoma; SUVmax = maximum standardized uptake value; TLG = total lesion glycolysis; WBMTV = whole body metabolic tumor volume; WBTLG = whole body total lesion glycolysis; HF; heterogeneity factor.

The forward stepwise multivariate Cox proportional hazards model indicated that nodal SUVmax (HR: 3.60) and WBMTV (HR: 3.15) were significant prognostic factors for DFS (Table 2).

### Comparison ROC for the prediction of tumor recurrence

**Figure 2:**
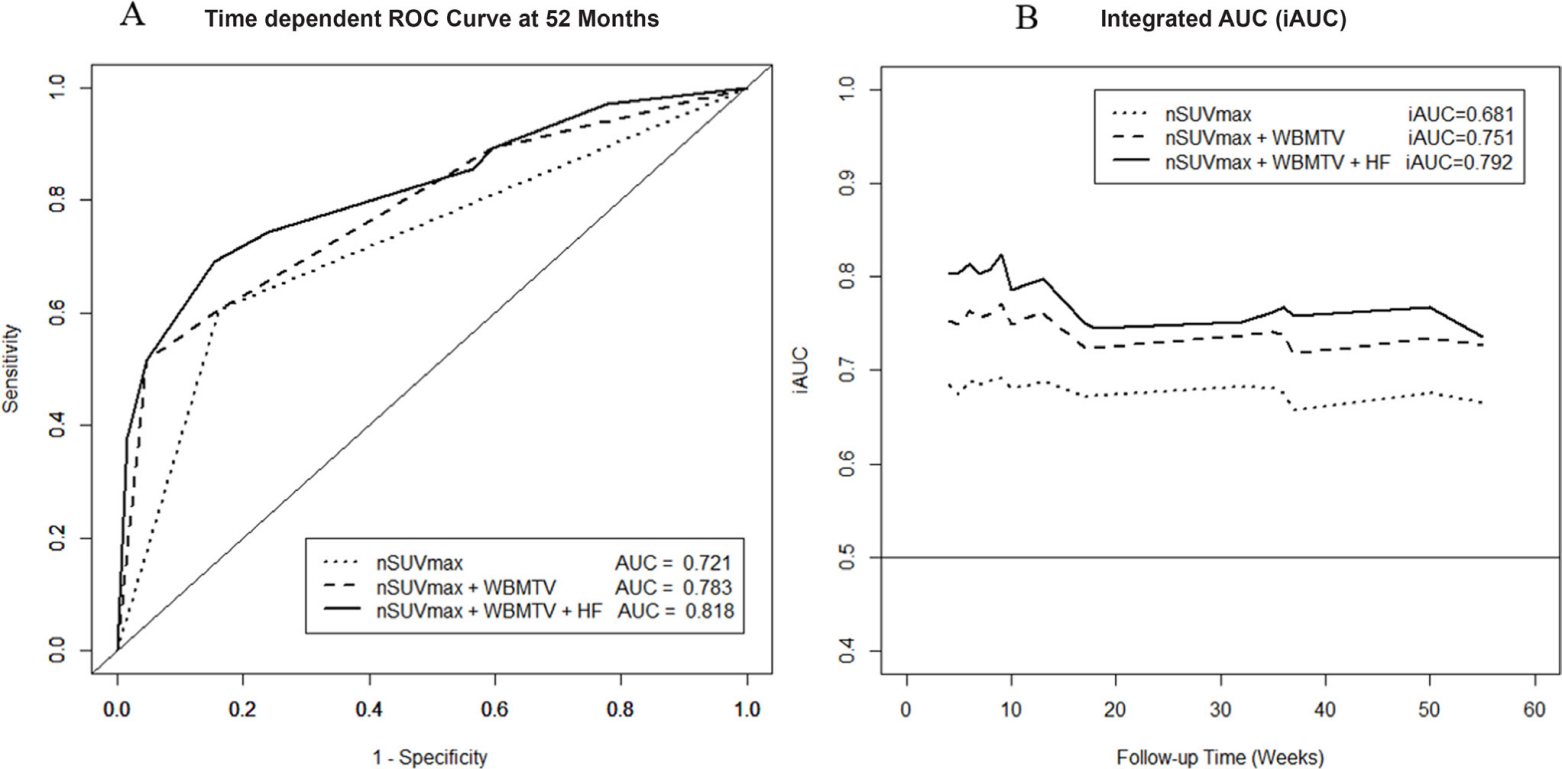
Additional value of heterogeneity factor for predicting tumor recurrence in the receiver operating characteristic (ROC) curve

To enhance discrimination of the recurrent group, HF was combined with nodal SUVmax and WBMTV. AUCs of nodal SUVmax, WBMTV, and HF were 0.732, 0.697, and 0.632, respectively (Table 3). When HF was combined with traditional metabolic parameters (nodal SUVmax and WBMTV), a significant improvement in discrimination for tumor recurrence was observed compared with nodal SUVmax alone (AUC: 0.817, *p* = 0.0028; Table 3 and Figure 2A). Furthermore, a net reclassification improvement showed significant improvement in the accuracy of risk prediction for DFS rates when HF was added to traditional metabolic risk factors (*p* = 0.0013; Table 3).

**Table 3:** Receiver operating characteristic (ROC) curve analysis for the prediction of tumor recurrence according to traditional metabolic parameters (nodal SUVmax and WBMTV), heterogeneity factor, and a combination of these parameters

Variables	ROC					Time-dependent ROC	
	AUC	95% CI	P1	P2 (IDI)	P3 (NRI)	iAUC	Difference (95% CI)
nSUVmax	0.732	0.632–0.833				0.681	
WBMTV	0.697	0.586–0.773				0.634	
HF	0.632	0.526–0.739				0.621	
nSUVmax+ WBMTV	0.811	0.728–0.895	0.0046	0.1275	0.0013	0.751	0.073 (0.033–0.133)
nSUVmax+ HF	0.781	0.681–0.881	0.0980	0.1677	0.0075	0.727	0.045 (0.006–0.103)
nSUVmax+ WBMTV+ HF	0.817	0.734–0.901	0.0028	0.1310	0.0013	0.792	0.111 (0.058–0.184)

AUC = area under curve; P1, P2, P3 = *p*-value for AUC between itself and nSUVmax; IDI = integrated discrimination improvement; NRI = net reclassification improvement; iAUC = integrated time dependent AUC; nSUVmax = nodal maximum standardized uptake value; WBMTV = whole body metabolic tumor volume; HF = heterogeneity factor.

When time-dependent ROC was used, integrated time-dependent AUC (iAUC) was 0.681 for nodal SUVmax, 0.634 for WBMTV, and 0.621 for HF. A combination of nodal SUVmax, WBMTV, and HF improved iAUC to 0.792, and the difference in iAUC compared with nodal SUVmax alone was 0.111 (95% CI, 0.058–0.184; Table 3). The iAUC value of the combination of nodal SUVmax, WBMTV, and HF varied with time, but remained higher than the combination of nodal SUVmax and WBMTV or HF (Figure 2B).

### DFS curve for combination of risk factors (nodal SUVmax, WBMTV, and HF)

**Figure 3:**
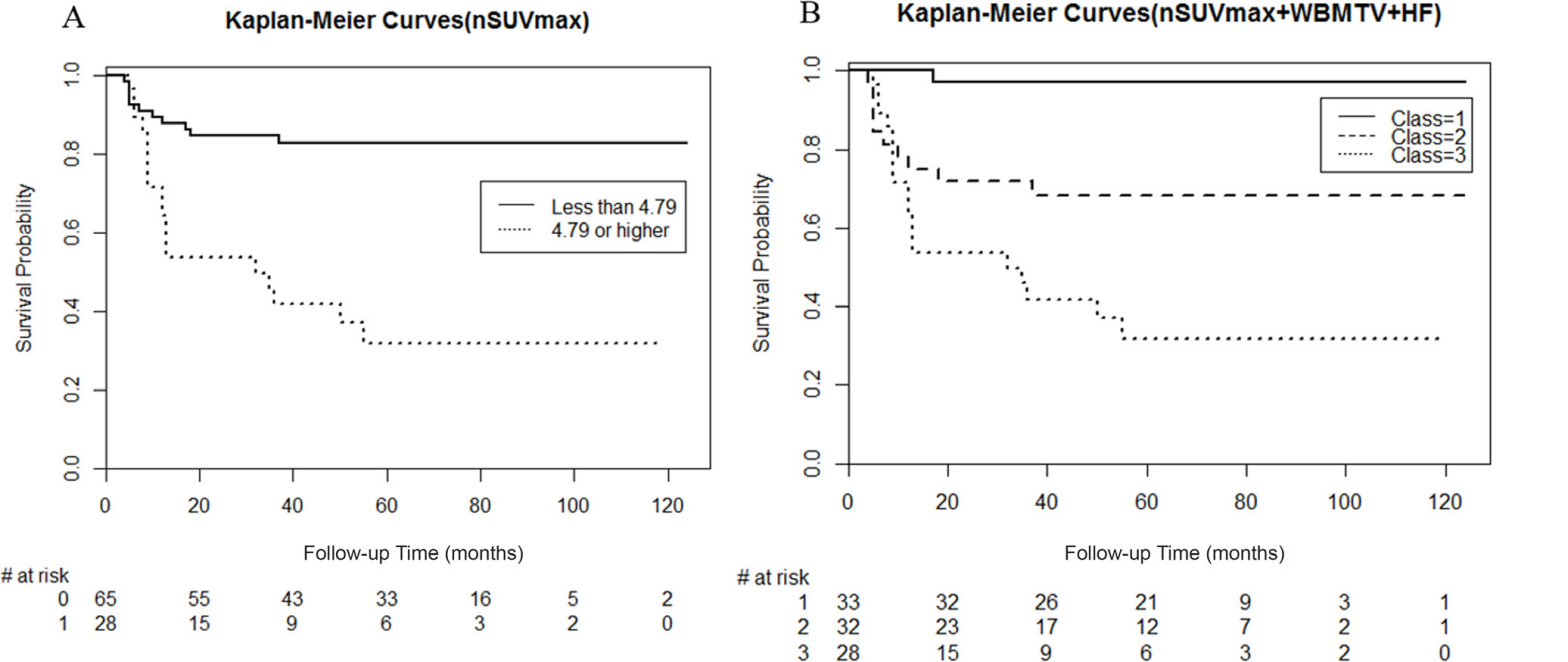
Kaplan-Meier survival plots of disease-free survival according to combined risk factors; (**A**) nodal maximum standardized uptake value only and (**B**) nodal maximum standardized uptake value, whole body metabolic tumor volume, and heterogeneity factor.

The risk factors were defined as nodal SUVmax, WBMTV, and HF. The weights’ factors of nodal SUV, WBMTV, and HF were 1.3, 1.0, and 0.25, respectively. These values were used to divide into three subgroups for a more detailed prognostic classification that included a combination of nodal SUVmax, WBMTV, and HF. The Kaplan-Meier analysis revealed that DFS significantly differed in subsets categorized based on nodal SUVmax alone (Figure 3A) and a combination of nodal SUVmax, WBMTV, and HF (Figure 3B). When using a combination of nodal SUVmax, WBMTV, and HF, the model fit is better than when nodal SUVmax is used alone (nodal SUVmax: Log-rank = 20.42, *p* = 6.2 × 10^–6^, combination nodal SUVmax, WBMTV and HF: Log-rank = 25.65, *p* = 4.1 × 10^–7^).

## DISCUSSION

The tumor microenvironment is inherently heterogeneous and is related to the variation in tumor responsiveness to treatment [23], degree of vascularity, hypoxia, proliferation rate [24], energy metabolism, and gene expression [25]. Tumors undergoing rapid proliferation often outgrow the existing vasculature, resulting in intratumoral hypoxia [26]. This adds complexity to the tumor biology and makes treatment planning difficult [27]. Cervical cancer is an example of tumors that show heterogeneity related to hypoxia and variation in response to treatment [23]. Moreover, the adaptation of tumors to hypoxia makes them more resistant to current cervical cancer therapies, including CCRT. The measurement of texture indices from tumor ^18^F-FDG PET/CT images has been recently proposed as an adjunct to predict tumor response to therapy.

We hypothesized that metabolic heterogeneity of cervical cancer could be correlated with known prognostic metabolic PET parameters (SUVmax, MTV, and TLG). According to our results, MTV was the factor most closely correlated with HF among the metabolic PET parameters. There was no correlation between SUVmax and HF. These findings suggest that intratumoral heterogeneity, which reflects varying tumoral metabolic characteristics, may correlate more with tumor size than with glucose metabolism in the tumor.

In this study, recurrence-free survival was shorter in patients with high HF, compared with patients with low HF. However, HF did not show superiority over standard metabolic parameters, including SUVmax, MTV, and TLG. Heterogeneity of FDG uptake within the tumor could be associated with more aggressive behavior and poorer response to treatment [12, 28]. In cervical cancer, a few previous studies have demonstrated that intratumoral FDG metabolic heterogeneity may be a useful predictor for tumor recurrence. At first, a study by Kidd et al. [14] reported that the intratumoral FDG metabolic heterogeneity on pre-treatment ^18^F-FDG PET/CT predicted the risk of lymph node involvement, pelvic recurrence, and response to therapy in patients with cervical cancer treated with CCRT. A study by Chung et al. [29] showed that preoperative intratumoral FDG heterogeneity was significantly associated with cervical cancer recurrence. However, that study included only early-stage cervical cancer with a 3.2-cm median value of primary tumor diameter (range 0.5–9.5 cm). A study by Brooks et al. [30] suggested that the inclusion of tumor volumes below 45 cm^3^ can profoundly bias comparisons of intratumoral uptake heterogeneity metrics derived from data from the current generation of whole-body ^18^F-FDG PET scanners. Therefore, intratumoral heterogeneity from early stage cervical cancer is not bias free. Recently, a study by Ho et al. [15] demonstrated that the heterogeneity of intratumoral FDG distribution may be an important predictor for overall survival in patients with bulky cervical cancer (≥ 4 cm) treated with CCRT. However, they included only 44 patients with bulky cervical cancer. Due to study limitations related to sample size and population, the exact role of intratumoral metabolic heterogeneity on pre-treatment ^18^F-FDG PET/CT in predicting tumor recurrence has not been fully investigated in patients with locally advanced cervical cancer.

ROC curves are a popular method for displaying the sensitivity and specificity of a continuous diagnostic marker for a binary disease variable. Analyzing differences in the AUC is a common method of comparing two models for prognostic risk prediction. To overcome the limitations of ROC curves, a study by Pencina et al. [21] introduced the integrated discrimination improvement and net reclassification improvement. The integrated discrimination improvement measures the ability of the new model to improve the average sensitivity without sacrificing average specificity. The net reclassification improvement measures the correctness of reclassification of patients based on their predicted probabilities of events using the new model with the option of imposing meaningful risk categories. Oncologic outcomes of cancer patients are time-dependent, and ROC curves that vary as a function of time may be more appropriate [22]. Therefore, we reconfirmed the additional prognostic value of HF using time-dependent ROC curves. In this study, nodal SUVmax was the single most independent prognostic factor for tumor recurrence. However, combining nodal SUVmax and WBMTV with HF improved the prognostic ability for predicting tumor recurrence compared with nodal SUVmax alone. A combination of nodal SUVmax, WBMTV, and HF improved the discrimination for tumor recurrence compared with nodal SUVmax alone not only in conventional ROC curves but also in net reclassification improvement and time-dependent ROC curves. Furthermore, the Kaplan-Meier survival curve analysis indicated that a risk group using a combination of nodal SUVmax, WBMTV, and HF was more suitable for dividing a risk group of recurrence compared to using nodal SUVmax alone. When using a combination of nodal SUVmax, WBMTV, and HF, patients with very low probability of recurrence were distinguished (Figure 3B). Therefore, HF may be a useful additional prognostic biomarker to improve the prognostic value of traditional metabolic parameters on ^18^F-FDG PET/CT.

Our study has some noteworthy limitations. First, it is a retrospective study with a limited number of patients. Second, the spatial resolution of FDG PET/CT scanning might affect the accuracy of the HF of small-sized tumors. Third, the use of two types of PET scanners in this study might have affected the values of quantitative metabolic parameters measured on FDG PET/CT. However, we performed an image analysis on a single workstation to minimize the effect of the use of different scanners. Finally, the intratumoral metabolic parameters on ^18^F-FDG PET/CT scans can be represented by various methods. The parameter of intratumoral metabolic heterogeneity is not well standardized. Furthermore, SUV measurements and region of interest alignments may have a few differences depending on user experience. The two nuclear medicine physicians who analyzed the PET images have more than 10 years of experience in PET image analysis and SUV measurements. Moreover, the ROI alignments were automatically obtained by using the volume viewer software. The intraobserver or interobserver variability was not significant (Supplementary Table 1).

Despite these limitations, our study offers some unique and significant findings and it differs from previous studies in several aspects. For example, we enrolled only patients with locally advanced cervical cancer with a FIGO stage > IIB. Moreover, we compared the prognostic values of HF and traditional metabolic parameters in patients with cervical cancer. Nodal SUVmax was the single most powerful predictive factor for DFS among the pre-treatment variables. HF did not show superiority over the traditional metabolic parameters. However, when nodal SUVmax was combined with HF, the predictive value for tumor recurrence was improved. Moreover, HF was correlated with only volume-associated metabolic parameters (MTV and TLG) and not with primary tumor SUVmax.

## CONCLUSIONS

Among the metabolic parameters measured with ^18^F-FDG PET/CT, HF highly correlated with the MTV in patients with locally advanced cervical cancer with a FIGO stage > IIB. No superiority of HF over the traditional metabolic parameters in terms of prognostic value for predicting tumor recurrence was observed in this study. Nevertheless, HF may be a valuable additional prognostic biomarker to improve the prognostic value of traditional metabolic parameters on ^18^F-FDG PET/CT.

## SUPPLEMENTARY MATERIALS FIGURES AND TABLES



## Abbreviations

CCRT: concurrent chemoradiotherapy
CI: confidence interval
DFS: disease-free survival
^18^F-FDG PET/CT: F-18 fluorodeoxyglucose positron emission tomography/computed tomography
FIGO: Advanced International Federation of Gynecology and Obstetrics
HF: heterogeneity factor
HR: hazard ratio
SUVmax: maximum standardized uptake value
MTV: metabolic tumor volume
ROC: receiver operating characteristic
TLG: total lesion glycolysis
WB: whole body
