# Supplementary material for: Prognostic value of intratumoral metabolic heterogeneity on F-18 fluorodeoxyglucose positron emission tomography/computed tomography in locally advanced cervical cancer patients treated with concurrent chemoradiotherapy
Source: Oncotarget. 2017 Jun 28;8(52):90402–12. doi: 10.18632/oncotarget.18769 (PMC5685760; doi:10.18632/oncotarget.18769)
Supplement: Supplementary file 1 [file oncotarget-08-90402-s001.pdf]

Prognostic value of intratumoral metabolic heterogeneity on F-18 fluorodeoxyglucose positron emission tomography/computed tomography in locally advanced cervical cancer patients treated with concurrent chemoradiotherapy

Supplementary Materials

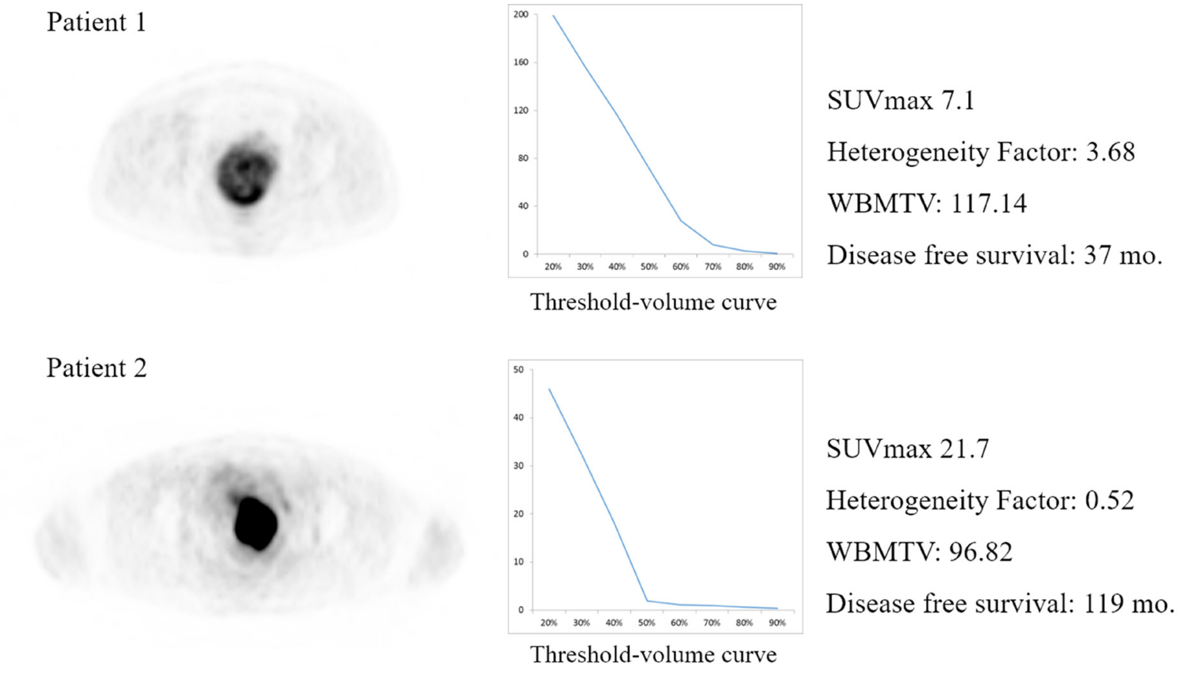

$$\text{heterogeneity} = H\ (\mathrm{d}V/\mathrm{d}T)$$

Supplementary Figure 1: Measurement of heterogeneity factor. Axial image of ^{18}F-FDG PET, threshold-volume curve, PET parameters and disease-free survival of two patients diagnosed cervical cancer and treated with concurrent chemoradiotherapy.

Supplementary Table 1: The intraclass correlation coefficient for intraobserver and interobserver variability of measurement of heterogeneity factor

		Intraobserver Agreement	Interobserver Agreement
Factors	No. of reader	HF measurement	HF measurement
Primary tumor	2	0.991 (0.987–0.994)	0.990 (0.985–0.993)

Note: Numbers in parentheses are the 95% CIs on the basis of 93 samples.
HF: heterogeneity factor
